# Involvement of the NF-κB signaling pathway in proliferation and invasion inhibited by Zwint-1 deficiency in Pancreatic Cancer Cells

**DOI:** 10.7150/jca.46173

**Published:** 2020-07-25

**Authors:** Jae Hyeong Kim, Yuna Youn, Jong-chan Lee, Jaihwan Kim, Jin-Hyeok Hwang

**Affiliations:** 1 Department of Internal Medicine, Seoul National University Bundang Hospital, Seongnam-si, Gyeonggi-do, 13620, Republic of Korea.; 2 Department of Internal Medicine, Seoul National University College of Medicine, Seoul, 03080, Republic of Korea.

**Keywords:** Zwint-1, NF-κB, CCAN, KMN, RZZ, pancreatic cancer, cancer biology

## Abstract

Pancreatic cancer (PC) is an intractable cancer that is difficult to diagnose early and has a 5-year survival rate of less than 8%. ZW10-interacting kinetochore protein (*ZWINT*) is a crucial gene that contributes to chromosome instability and is essential for spindle assembly and kinetochore-microtubule attachment during meiosis and mitosis. However, the mechanism through which Zwint-1 promotes PC progression is yet to be elucidated. Here, we report that Zwint-1 is highly expressed in clinical PC specimens (based on analysis of the Gene Expression Profiling Interactive Analysis database) and various PC cell lines. Importantly, Zwint-1-deficient PC cells showed reduced nuclear factor-kappa B (NF-κB) (Ser536) phosphorylation along with inhibited proliferation and colony formation due to downregulation of NF-κB-regulated genes such as *CCND1*, *cIAP1/2*, and *XIAP*. In addition, Zwint-1-deficient PC cells showed reduced invasion and migration abilities, and decreased expression levels of the metalloproteinases MMP2 and MMP9. Furthermore, Zwint-1 deficiency arrested the PC cell cycle at the G_2_/M phase because the chromosomes failed to segregate properly, and the apoptosis rate in these cells gradually increased, accompanied by increased caspase-3 activation and anti-poly (ADP ribose) polymerase cleavage. Apoptosis caused by Zwint-1 deficiency was demonstrated to occur through caspase-dependent pathways based on experiments involving treatment with a pan-caspase inhibitor (Z-VAD-Fmk). Thus, Zwint-1 contributes to cell growth, invasion, and survival through NF-κB signaling pathways, suggesting that it could serve as a PC biomarker and new therapeutic target.

## Introduction

All eukaryotic cells must precisely replicate their genomes during mitosis and then distribute them to newly forming daughter cells. For proper chromosome segregation, sister chromosomes must bind to the microtubules extending from opposing spindle poles through the kinetochore, a dynamic multi-protein assembly on the centromere 1-4. Kinetochores consist of hundreds of conserved proteins, which are divided into major structural sub-complexes such as the constitutive centromere-associated network (CCAN), KNL1-Mis12-Ndc80 (KMN) network, and ROD-Zwilch-ZW10 (RZZ) complexes 1-3, 5, 6. The CCAN, which has several kinetochore subunits, is further assembled into a CENP-A nucleosome 7 and is constitutively localized to the centromere throughout the cell cycle 6, 8. The CCAN acts as a foundation for kinetochore assembly and is a connector for centromeric chromatin, while the KMN network forms a complex with the microtubule-binding site of the kinetochore 8. CENP-C binds to Ndc80 complexes through interactions with KNL1 and Mis12 complexes, and CENP-T is directly linked to Ndc80, contributing to KMN network localization 9. Ndc80 complexes also directly bind microtubules 2. RZZ is required for dynein/dynactin recruitment 10-12 and spindle assembly checkpoint activation at the kinetochore 13.

ZW10-interacting kinetochore protein 1 (Zwint-1) was initially identified as a protein that interacts with Zeste White 10 (ZW10) by a yeast two-hybrid screen, and was subsequently demonstrated to be a kinetochore component that plays an important role in spindle assembly and kinetochore-microtubule attachment during meiosis and mitosis 14-16. Zwint-1 can directly interact with components of the KMN complex, specifically Ndc80 and Mis12, and acts as a bridge between the RZZ and KMN complexes required for kinetochore formation and spindle checkpoint activity 17. Zwint-1 is also a mitotic checkpoint component required for the stable association of CENP-F and dynamitin with the kinetochore to ensure accurate chromosome segregation 15. Recent studies have suggested that Zwint-1 could be a potential cancer biomarker 18, 19 given its high expression levels in several human malignancies such as prostate cancer, ovarian cancer, bladder cancer, lung cancer, hepatocellular carcinoma, and pulmonary adenocarcinoma 18, 20-25. Moreover, knockdown of Zwint-1 has been shown to inhibit the proliferation, migration, invasion, and colony formation of lung cancer cell, and to enhance cell apoptosis 23.

However, little is known about the role of Zwint-1 in pancreatic cancer (PC). In this study, we investigated the expression of Zwint-1 in tissues from patients with PC in The Cancer Genome Atlas (TCGA) database and in PC cell lines, and evaluated the effects of Zwint-1 on PC cell tumorigenesis and progression using a Zwint-1 knockdown system *in vitro*.

## Methods

### Database analysis

Expression levels of *ZWINT, CCAN* (*CENP-A, CENP-C, CENP-T*)*, KMN* (*KNL1, Mis12, Ndc80*), and *RZZ* (*ROD, Zwilch, ZW10*) complexes in 179 PC and 171 normal pancreas tissues were compared by Gene Expression Profiling Interactive Analysis (GEPIA) (http://gepia.cancer-pku.cn/) 26 based on patient data from TCGA 27 and normal tissue samples from Genotype-Tissue Expression (GTEx) 28, 29. The TCGA dataset is based on RNA-sequencing performed on 179 PC tissues from 178 PC patients, including 178 primary tissues and one metastasis tissue. The expression was determined as transcripts per million (TPM), and gene expression levels were calculated using a log_2_ (TPM+1) scale for comparison. The cutoff values were |log_2_ fold change (FC)| of 1 and P value < 0.01. The overall survival (OS) of patients with PC was extracted from TCGA data and compared with the expression levels of the Zwint-1-related genes.

### Cell culture and small interfering RNA (siRNA) knockdown

The human PC cell lines MIA PaCa-2 [American Type Culture Collection (ATCC) CRL-1420; ATCC, Manassas, VA, USA] and PANC-1 (ATCC CRL-1469) were grown in high-glucose Dulbecco's modified Eagle's medium (DMEM). The human pancreatic ductal adenocarcinoma (PDAC) cell lines AsPC-1 and Capan-1 were grown in RPMI medium. Noncancerous immortalized human pancreatic duct epithelial (HPDE) cells, obtained from Joo Kyung Park, MD (Samsung Medical Center, Seoul, South Korea), were grown in defined K-SFM medium. All cell culture media contained 10% fetal bovine serum (FBS), 100 U/mL penicillin and 100 μg/mL streptomycin (Gibco, Life Technologies, Grand Island, NY, USA). To block Zwint-1 expression, the cells were transfected with specific siRNA using Lipofectamine RNAiMAX transfection reagent (Invitrogen, Carlsbad, CA, USA) and then tested for efficacy according to the manufacturer's instructions. Two different Zwint-1 siRNAs were prepared 30. The two Zwint-1 siRNAs and control siRNA were purchased from Cosmo Bio Co., Ltd. (Tokyo, Japan).

### Cell proliferation and viability assay

After adhesion for 24 h, the cells were transfected with Zwint-1 siRNA or control siRNA and incubated for 3 or 7 d. For cell proliferation and viability assays, MIA PaCa-2 and PANC-1 cells were seeded into 12-well plates at a density of 1 × 104 cells/well or 8 × 104 cells/well and tested using 3-(4,5-dimethylthiazol-2-yl)-2,5-diphenyltetrazolium bromide (MTT) solution. For colony formation assays, the cells were seeded into 6-well plates at a density of 500-1000 cells/well and incubated for 14 d. The colonies were then fixed in 100% methanol, stained with 10% crystal violet, and counted. Each assay was performed in triplicate.

### Wound-healing assay

The motility of Zwint-1 siRNA and control siRNA-transfected cells was examined by a wound-healing assay. The cells were seeded in 6-well plates at 5 × 10^5^ cells per well. After 24 h, wounds were created in cell monolayers in each well using a P1000 pipette tip. The cells were then rinsed once with phosphate-buffered saline (PBS) and cultured for 24 h or 72 h. Cell motility was expressed as the cell migration rate.

### Cell migration and invasion assays

Migration and invasion abilities under Zwint-1 deficiency were assessed using Transwell plates (Corning, Corning, NY, USA). For the cell migration assay, 5 × 10^4^ cells in 200 μL of 1% serum DMEM were seeded directly into each well of the Transwell chambers with 8-μm pore membranes. For the cell invasion assay, the cells were seeded in the Transwell chambers coated with Matrigel (Corning), and medium containing 10% FBS was added to the lower chamber. After incubation for 24 h, the medium in the upper chamber was removed, and the cells were fixed and stained using the Differential Quik Stain Kit. The cells adhering to the upper surface of the membrane were removed using a cotton applicator. The number of cells on the lower side of the membrane was counted. Each experiment was performed in triplicate.

### Western blotting

Proteins were extracted from whole cells using 1× RIPA buffer, and protein concentrations were determined using a BCA Protein Assay Kit (Pierce, Rockford, IL, USA). Protein extracts were resuspended in 5× sample buffer [50 mM Tris (pH 6.8), 100 mM dithiothreitol, 2% sodium dodecyl sulfate (SDS), 0.1% bromophenol blue, and 10% glycerol], boiled for 5 min, and subjected to SDS-polyacrylamide gel electrophoresis on 8-15% gels. Separated proteins were transferred to transblot nitrocellulose membranes (Schleicher & Schuell, Keene, NH, USA), and membranes were blocked in 5% skim milk with TBST [10 mM Tris (pH 8.0), 150 mM NaCl, 0.05% Tween 20] and incubated with the following primary antibodies: anti-Zwint-1 (GeneTex), anti-p-NF-κB (p65) (Ser536) (Cell Signaling Technology, Danvers, MA, USA), anti-NF-κB (p65) (Cell Signaling Technology), anti-cyclin D1 (Cell Signaling Technology), anti- cellular inhibitor of apoptosis 1/2 (cIAP1) (Cell Signaling Technology), anti-cIAP2 (Cell Signaling Technology), anti-X-linked inhibitor of apoptosis (XIAP) (Cell Signaling Technology), anti-MMP2 (Cell Signaling Technology), anti-MMP9 (Cell Signaling Technology), anti-cyclin A2 (Cell Signaling Technology), anti-p-histone H3 pS10 (Abcam, Cambridge, MA, USA), anti-caspase-3 (Cell Signaling Technology), anti-poly (ADP ribose) polymerase (PARP; Cell Signaling Technology), and anti-β-actin (Cell Signaling Technology). Protein expression was detected based on chemiluminescent signals activated by SuperSignal West Pico Chemiluminescent Substrate (Pierce, Thermo Scientific, USA) after reaction with horseradish peroxidase-tagged secondary antibodies (Jackson Immunoresearch Laboratories, West Grove, PA, USA).

### Fluorescence-activated cell sorting (FACS) analysis

For cell cycle and DNA content analysis, cultured cells were incubated in trypsin-ethylenediaminetetraacetic acid at 37°C in an atmosphere containing 5% CO_2_, collected by centrifugation, and washed once with 1× PBS. The cells were centrifuged, supernatants were removed, and then the cells were stained with 50 μg/mL propidium iodide (PI; Sigma-Aldrich, St. Louis, MO, USA), along with 100 U ribonuclease A from the bovine pancreas (Sigma-Aldrich). FACs analysis was performed with a FACSCalibur instrument (BD Biosciences, San Diego, CA, USA) according to standard protocols.

Apoptosis was also analyzed by FACS using fluorescein isothiocyanate (FITC)-conjugated annexin V (BD Biosciences, San Diego, CA, USA) and PI (Sigma-Aldrich) staining. The analysis was performed with a FACSCalibur (BD Biosciences) instrument according to the standard protocol. Apoptosis was blocked with the pan-caspase inhibitor Z-VAD-FMK (20 µM; R&D Systems, Minneapolis, MN, USA).

### Immunofluorescence

Cells were grown on Thermo Scientific Nunc Lab-Tek II Chamber Slides, permeabilized with 0.5% Triton X-100 for 1 min, and fixed with 4% paraformaldehyde for 10 min. The fixed cells were incubated for 1 h at room temperature with blocking solution (1% bovine serum albumin) and then incubated overnight at 4°C with anti-CREST (Immuno Vision Technologies, Brisbane, CA) and anti-α-tubulin (Abcam, Cambridge, MA, USA) primary antibodies. The cells were then incubated with secondary antibodies and 100 ng/mL DAPI for 3 h. Samples were mounted in Prolong Gold Antifade reagent (Invitrogen) and viewed under a confocal microscope (Zeiss LSM710 with ZEN software).

### Statistical analysis

All data are presented as the mean ± standard error of the mean (SEM) values from three independent experiments. Results were statistically analyzed using GraphPad Prism (version 5.0, GraphPad Software Inc., San Diego, CA, USA) using one-way or two-way analysis of variance followed by Bonferroni's multiple comparison tests.

## Results

### Zwint-1 expression is upregulated in PC patient tissues and PC cell lines

**Figure 1:**
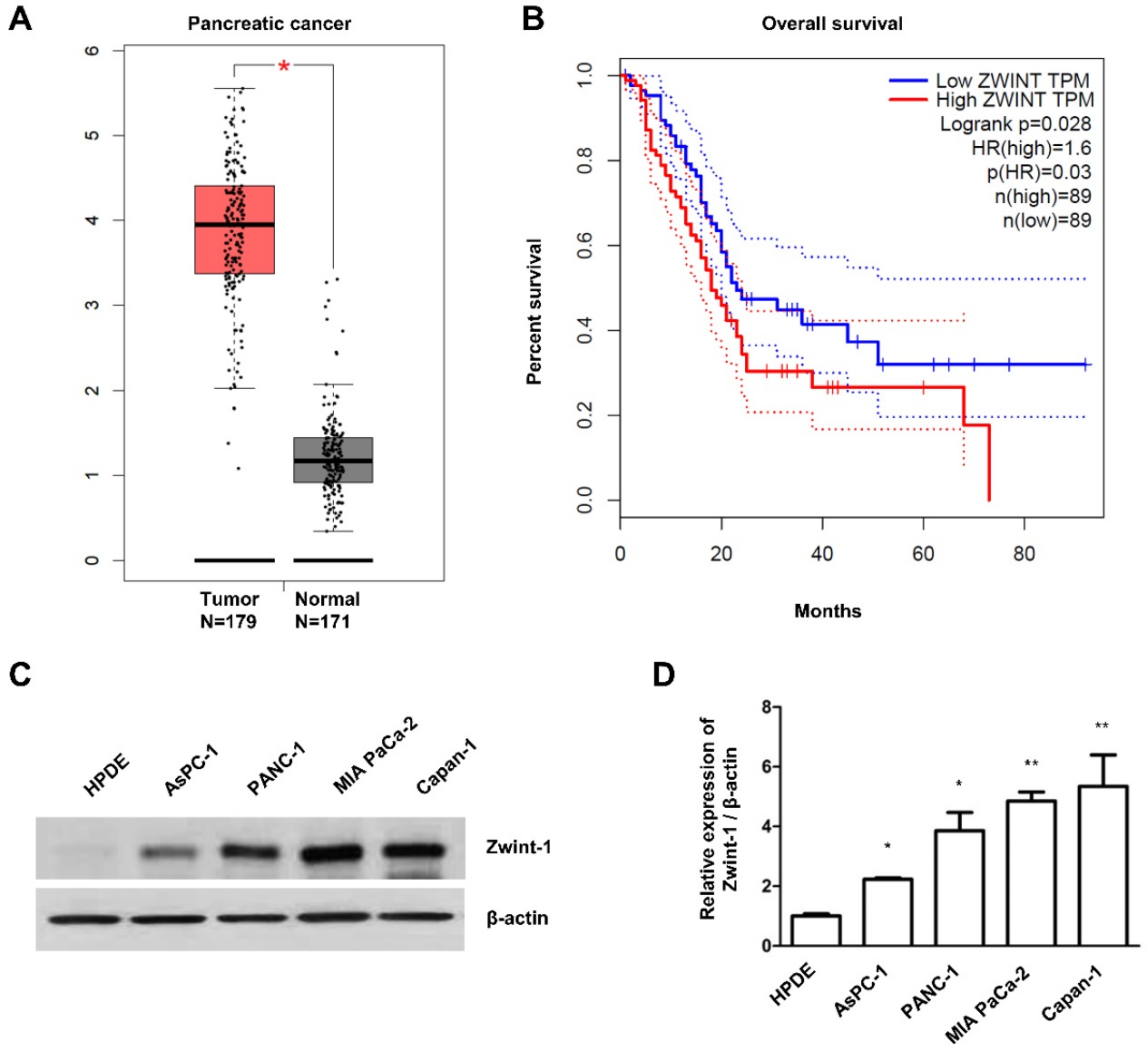
** Zwint-1 expression is elevated in PC tissues and cell lines. (A)** Zwint-1 expression levels in PC tissues (n = 179) and normal tissues (n = 171). **(B)** OS based on the TCGA database. Each dot represents the Zwint-1 expression level in one sample. The OS of PC patients with low or high Zwint-1 expression levels was analyzed by the Kaplan-Meier method and a log rank test. The high dotted line demarcates samples with higher expression levels than the median value of TPM (the high expression cohort), whereas the low dotted line demarcates samples with lower expression levels than the median value of TPM (the low expression cohort). Median values ​​are indicated by solid lines. *P < 0.005. TCGA, The Cancer Genome Atlas; TPM, transcripts per million; OS, overall survival; HR, hazard ratio. **(C)** Zwint-1 protein expression analyzed by western blotting in HPDE cells and different PC cell lines (AsPC-1, PANC-1, MIA PaCa-2, and Capan-1 cells). Cell lysates were immunoblotted with the indicated antibodies. **(D)** Levels of Zwint-1 protein expression in HPDE and PC cell lines. The Zwint-1/β-actin ratio was determined by densitometric analysis using ImageJ software. Error bars represent standard deviations of the means of three biological replicates. Values represent means ± SEMs. *P < 0.005, **P < 0.001. Results were analyzed by one-way analysis of variance followed by Bonferroni's multiple comparison tests.

We first confirmed the mRNA expression of the CCAN (*CENP-A, CENP-C, CENP-T*), KMN (*KNL1, Mis12, Ndc80*), and RZZ (*ROD, Zwilch, ZW10*) complexes, as well as *Zwint-1*, in clinical PC specimens from TCGA (n = 179) and normal tissues from GTEx data (n = 171) using the publicly available GEPIA database. Zwint-1 expression was elevated in tumors, whereas expression of the genes involved in the CCAN, KMN, and RZZ complexes was not (Fig. 1A and Supplementary Fig. 1A). In addition, the OS of PC patients was inversely proportion to the Zwint-1 expression level (Fig. 1A and B). Other clinical and demographic information about the patients is shown in Supplementary Fig. 2A-I, which was obtained from the cBioportal database. We also measured the level of Zwint-1 protein in various PC lines, including AsPC-1, PANC-1, MIA PaCa-2, and Capan-1 cells, by western blotting, which demonstrated higher levels in AsPC-1, PANC-1, MIA-PaCa-2, and Capan-1 cells compared to those in normal HPDE cells (Fig. 1C and 1D).

### Zwint-1 deficiency inhibits the colony formation and proliferation of PC cells via the nuclear factor kappa B (NF-κB) signaling pathway

**Figure 2:**
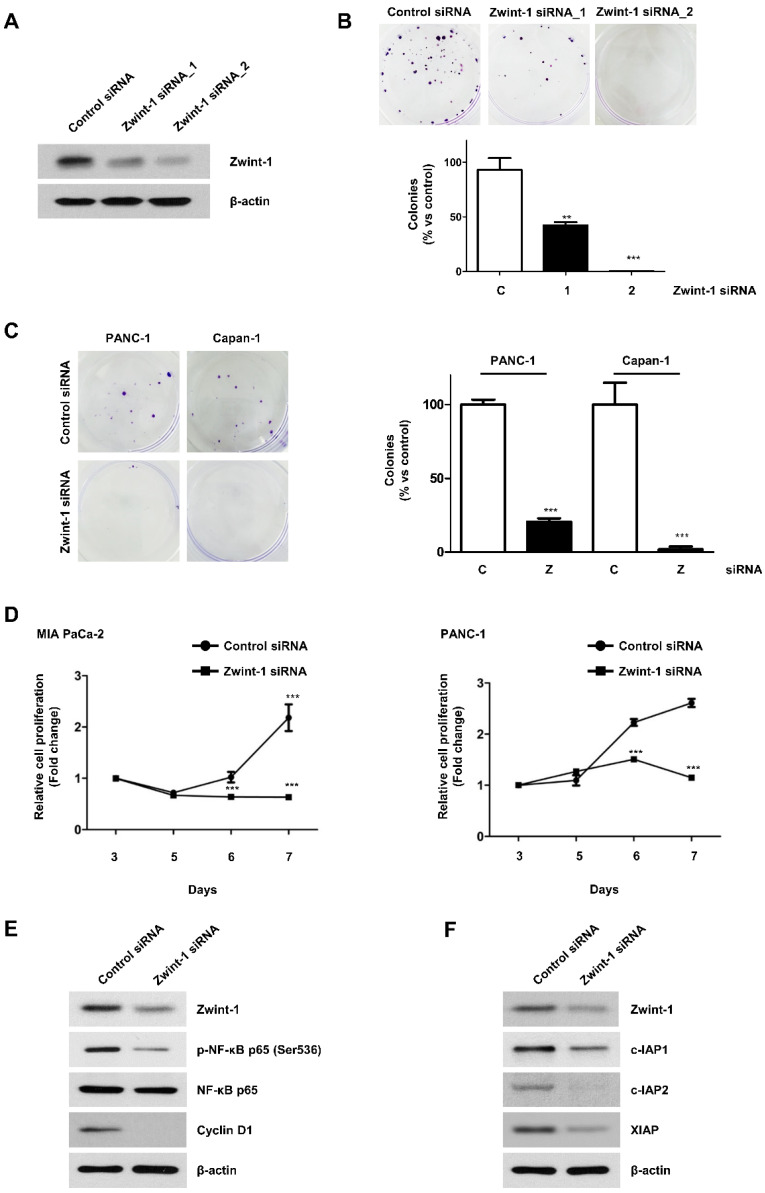
** Zwint-1-deficient PC cells show reduced proliferation and colony formation mediated by NF-κB signaling. (A)** MIA PaCa-2 cells were transfected with two different siRNA constructs to knock down Zwint-1 expression, and the expression levels of Zwint-1 protein were measured 48 h later by western blotting.** (B)** Colony formation assays on MIA PaCa-2 cells transfected with control or one of two Zwint-1 siRNAs (Zwint-1 siRNA_1 and siRNA_2). **(C)** Colony formation assays in PANC-1 and Capan-1 cells transfected with control siRNA and Zwint-1 siRNA_2. Values represent means ± SEMs. Results were analyzed using unpaired *t*-tests. ***P < 0.0001. **(D)** MTT assays to determine the proliferation of control and Zwint-1-deficient PC cells for 3 or 7 d. Curves were constructed based on biological triplicates with values expressed as means ± SEMs. ***P < 0.0001. Results were analyzed by two-way analysis of variance with Bonferroni's multiple comparison tests. **(E)** Western blotting to determine the levels of Zwint-1, p-NF-κB (Ser536), NF-κB p65, Cyclin D1, and β-actin in MIA PaCa-2 cells after transfection with control or Zwint-1 siRNA at 24 h. **(F)** Western blotting to determine the levels of Zwint-1, c-IAP1, c-IAP2, XIAP, and β-actin in MIA PaCa-2 cells after transfection with control or Zwint-1 siRNA at 48 h.

Between the two siRNAs tested, Zwint-1_2 had a greater effect on inhibiting Zwint-1 protein expression better (Fig. 2A), resulting in reduced colony formation in MIA PaCa-2 (Fig. 2B), PANC-1, and Capan-1 cells (Fig. 2C). Therefore, Zwint-1_2 siRNA was selected for use in subsequent experiments. The MTT assay showed that Zwint-1 deficiency significantly inhibited the proliferation of MIA PaCa-2 and PANC-1 cells for 3-7 d after siRNA transfection (Fig. 2D). Western blotting showed that the expression level of p-NF-κB (p65) (Ser536) was reduced due to Zwint-1 deficiency, whereas there was no difference in the expression level of NF-κB (p65) between Zwint-1-expressing and Zwint-1-deficient cells. In addition, the expression level of cyclin D1 (a target of NF-κB) was significantly reduced due to Zwint-1 deficiency in PC cells (Fig. 2E). Finally, the expression levels of cIAP1/2 and XIAP, targets of NF-κB involved in cell survival, were also markedly reduced in Zwint-1-deficient PC cells (Fig. 2F). These results indicated that Zwint-1 is involved in the proliferation and survival of PC cells at least partially through NF-κB signaling.

### Zwint-1 deficiency reduces the migration and invasion capacity of PC cells

**Figure 3:**
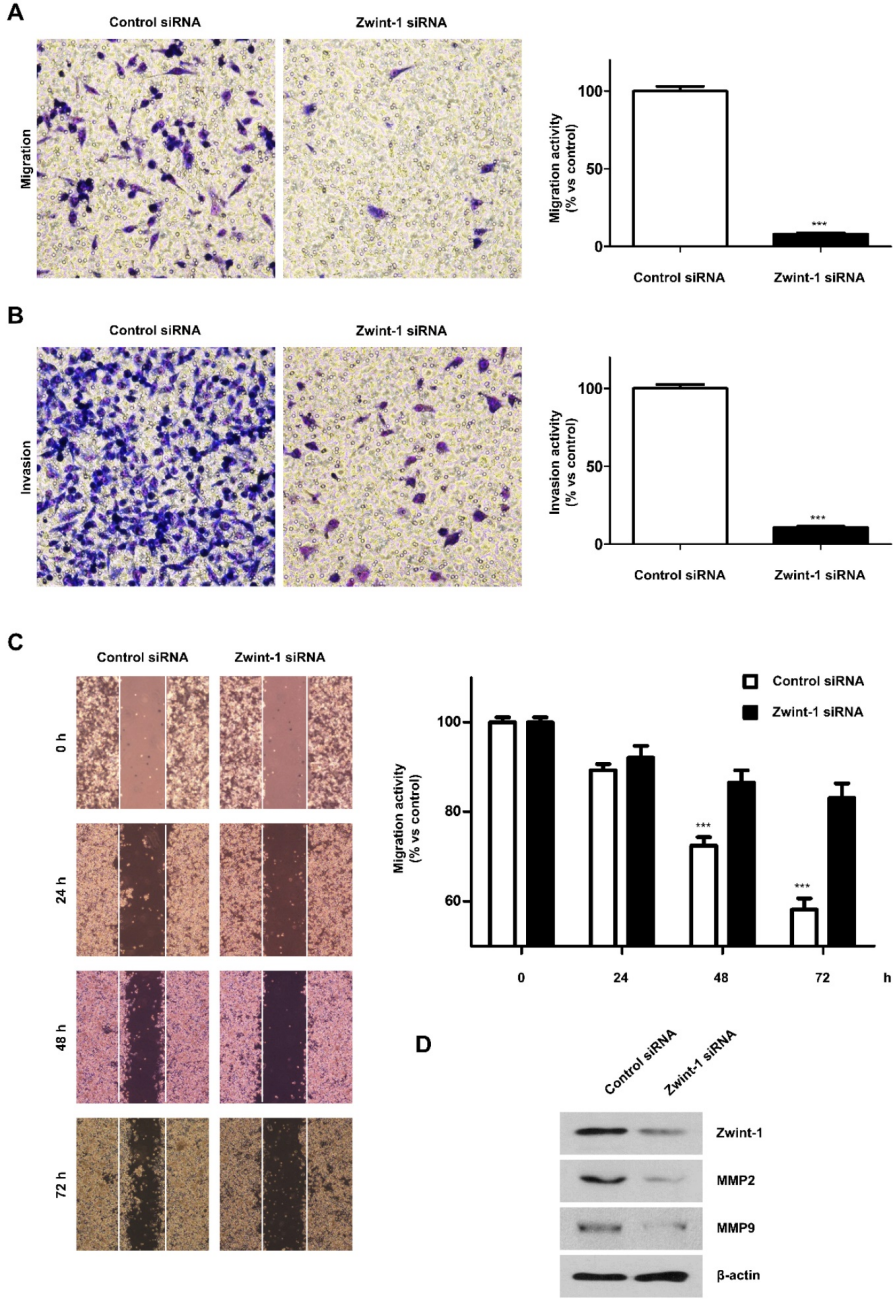
** Zwint-1-deficient PC cells show reduced migration, invasion, and wound healing abilities due to MMP2 and MMP9 inhibition. (A)** Migration and **(B)** invasion assays of MIA PaCa-2 cells transfected with control or Zwint-1 siRNA at 24 h. **(C)** Wound-healing assays of MIA PaCa-2 cells transfected with control or Zwint-1 siRNA for 24-72 h. Values represent means ± SEMs. Results were analyzed using unpaired *t*-tests. ***P < 0.0001. **(D)** Western blotting to determine the levels of Zwint-1, MMP2, MMP9, and β-actin in MIA PaCa-2 cells after transfection with control or Zwint-1 siRNA at 48 h.

After inhibiting PC cell growth by culturing under low FBS conditions, the number of migrating Zwint-1-deficient MIA-PaCa-2 cells was markedly reduced compared to that of control cells (Fig. 3A). Transwell assays further showed that the number of invading Zwint-1-deficient MIA-PaCa-2 cells was reduced compared to that of the control cells (Fig. 3B), and the wound-healing ability was also decreased in Zwint-1-deficient PC cells (Fig. 3C) further confirming reduced migration potential, particularly after 48 h. These results were supported by the reduced expression levels of MMP-2 and MMP-9 under Zwint-1 deficiency (Fig. 3D), indicating that Zwint-1 plays essential roles in migration and invasion by activating MMP2 and MMP9 expression in PC cells.

### Zwint-1-deficient PC cells are arrested in the G_2_/M phase leading to high rates of apoptosis

**Figure 4:**
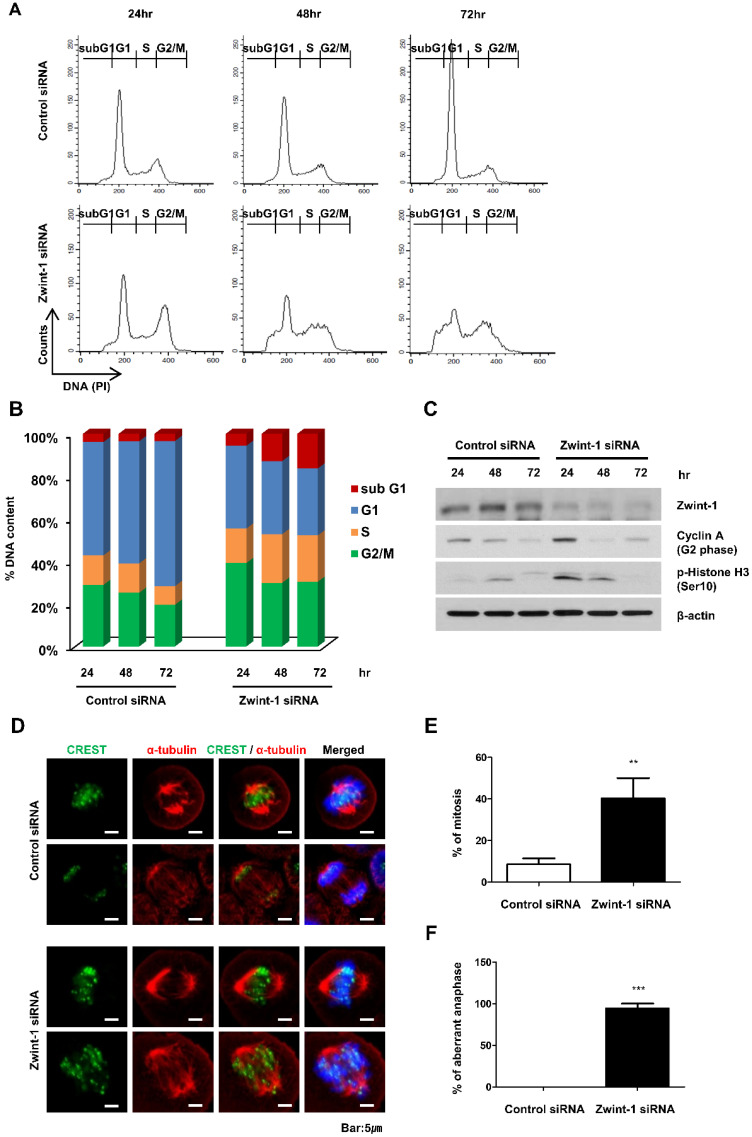
** Zwint-1-deficient PC cells are arrested in the G_2/_M phase and undergo apoptosis. (A)** MIA PaCa-2 cells were transfected with control or Zwint-1 siRNA and then subjected to flow cytometric analysis at 24, 48, and 72 h. The flow cytometry plots and data are representative of at least three separate experiments. **(B)** Percentages of cells in subG_1_ (apoptosis, red), G_1_ (blue), S (orange), and G_2_/M (green) phases. **(C)** Western blotting to determine the levels of Zwint-1, cyclin A2, p-histone H3, and β-actin in MIA PaCa-2 cells after transfection with control or Zwint-1 siRNA for 24, 48, and 72 h. **(D)** Spindle structures were visualized by immunofluorescent staining for CREST (green), a-tubulin (red), and DNA (blue). **(E)** Comparison of mitosis frequency in Zwint-1 siRNA-transfected cells versus control siRNA-transfected cells.** (F)** Comparison of aberrant anaphase frequencies in Zwint-1-trasnfected siRNA cells versus those in control siRNA-transfected cells. Values represent means ± SEMs. Results were analyzed using unpaired *t*-tests. **P < 0.001, ***P < 0.0001.

Flow cytometry to determine the proportion of cells in the subG_1_, G_1_, S, and G_2_/M phases of the cycle demonstrated an increased proportion of Zwint-1-deficient PC cells in G_2_/M compared to that in controls. In addition, subG_1_ populations (apoptotic cells) increased compared to those in the controls at 24 to 72 h, whereas the G_1_ cell population was decreased in Zwint-1-deficient PC cells in a concentration-dependent manner (Fig. 4A, 4B). Using western blotting, we also measured the expression of cyclin A2 (a regulator of the G_1_ phase) and p-histone H3 (Ser10) (a regulator of the G_2_/M phase transition) to determine the molecular mechanisms altered by Zwint-1 deficiency. After 24 h of Zwint-1 siRNA transfection, cyclin A2 and p-histone H3 (Ser10) levels were significantly increased in the Zwint-1-deficient PC cells (Fig. 4C). Immunofluorescence observations of the centromere markers CREST and alpha-tubulin demonstrated well-aligned chromatids separated into two poles with a centromere in control cells. By contrast, although the tubulin of Zwint-1-deficient PC cells was attached to the centromere, no normally dividing cells were observed (Fig. 4D-4F). These results indicated that Zwint-1-deficient PC cells were arrested in the G_2_/M phase because they could not divide.

### Apoptosis is increased in Zwint-1-deficient PC cells through caspase-dependent signaling

**Figure 5:**
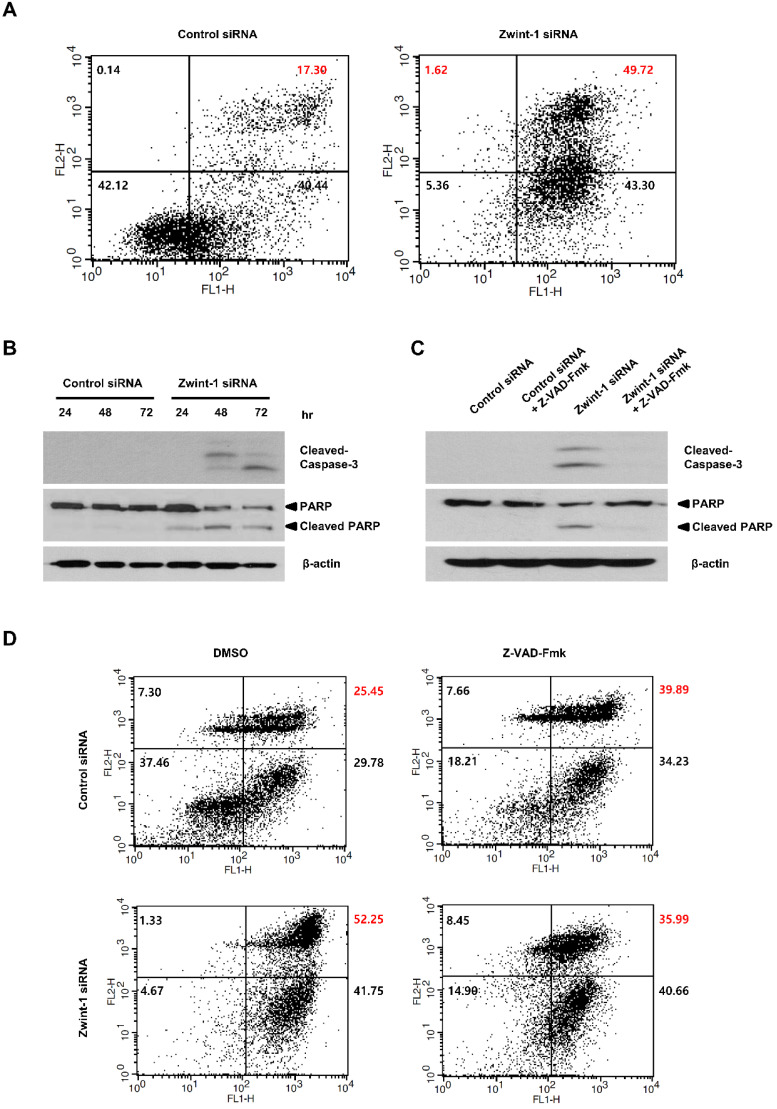
** Zwint-1-deficient PC cells undergo apoptosis induced by a caspase-dependent pathway. (A)** Apoptosis was measured by annexin V staining followed by flow cytometry. The lower left (LL) quadrant shows live cells, the lower right (LR) shows early-apoptotic cells, and the upper right (UR) shows late-stage apoptotic cells. **(B)** Western blotting to determine the levels of cleaved-caspase-3, PARP, and β-actin in MIA PaCa-2 cells after transfection with control or Zwint-1 siRNA for 24, 48, and 72 h. **(C)** Western blot analysis to determine the levels of caspase-3, PARP, and β-actin in MIA PaCa-2 cells after transfection with control or Zwint-1 siRNA with or without Z-VAD-Fmk (20 μM) treatment for 72 h. **(D)** Apoptosis measured by annexin V staining followed by flow cytometry. Cells were treated with Z-VAD-Fmk (20 µM) for 72 h.

To determine whether Zwint-1-deficient PC cells were lost through apoptosis or necrosis, we double-stained the cells with annexin V-FITC and PI and quantified phenotypic changes in apoptotic cells. FACS analysis showed that the proportion of the late-apoptosis (annexin V-FITC-positive and Annexin V-FITC/PI-positive) population was significantly higher in Zwint-1-deficient cells compared to that in control cells at 72 h (Fig. 5A, upper right quadrant). The levels of caspase-3 and cleaved PARP proteins increased in Zwint-1-deficient PC cells compared to those of controls (Fig. 5B), further confirming a role of apoptosis in the observed cell death. These results are consistent with the increase in the subG_1_ population in Zwint-1-deficient PC cells. Treatment of Zwint-1-deficient PC cells with 20 μM of the pan-caspase inhibitor Z-VAD-Fmk reduced the rate of apoptosis (Fig. 5C), and also reduced the levels of caspase-3 activation and PARP cleavage increased underZwint-1 deficiency (Fig. 5D). These results indicated that increased apoptosis due to Zwint-1 deficiency occurs through caspase-dependent signaling pathways.

## Discussion

Zwint-1 is not only necessary for normal cell division during mitotic metaphase but is also highly expressed in various carcinomas. In this study, we showed that Zwint-1 is highly expressed in PC cells and tissues, and promotes the proliferation and invasion of PC cells through NF-κB signaling. In line with previous studies in patients with other cancer types 18, 20-25, Zwint-1 was found to be upregulated in PC tissues and cell lines, and patients with high Zwint-1 expression had lower OS than those with low Zwint-1 expression. Zwint-1-deficient PC cells exhibited suppressed colony formation, proliferation, migration, and invasion due to a reduction in NF-κB phosphorylation and the expression of related genes. These data support evidence that Zwint-1 knockdown inhibits lung cancer cell proliferation, migration, and invasion, as well as colony formation 23. Furthermore, Zwint-1-deficient PC cells showed induced G_2_/M arrest due to abnormal cell division, which promoted apoptosis through caspase-dependent pathways. These findings suggest that Zwint-1 might play an important role in the promotion and progression of PC. To the best of our knowledge, this is the first report showing that Zwint-1 contributes to PC cell progression through NF-κB signaling.

The NF-κB family of transcription factors includes RelA (p65), RelB, c-Rel, NF-κB1 (p50/p105), and NF-κB2 (p52/p100), which play important roles in inflammation, cell proliferation and differentiation, immune responses, and cancer 31-33. They share a Rel homology domain that regulates the development and progression of cancer by allowing the binding of NF-κB-specific DNA motifs 34. Masking nuclear position signals prevents NF-κB transcription factors from being translocated into the cell nuclei by NF-κB inhibitor (IκB); thus, NF-κB remains dormant and inactive in the cytoplasm 35. However, in most cancers, NF-κB is activated and is considered to be a major signal mediator that contributes to cancer development and progression by promoting cell proliferation, regulating apoptosis, stimulating angiogenesis, and increasing invasion and metastasis 36-38. In particular, NF-κB signaling pathways play an important role in the development and progression of PC and drug resistance 39. Indeed, we observed that Zwint-1-deficient PC cells inhibited temozolomide resistance (Supplementary Fig. 3A).

NF-κB also plays an essential role in controlling the transcription of genes such as cyclooxygenase-2 (*COX2*) and cyclin D1, which are important in the early and late stages of aggressive cancers; *cIAP-1/2, XIAP*, and cellular FLICE inhibitory protein (*FLIP*), which are important genes that encode apoptosis suppressor proteins; and *MMP2*, *MMP9*, and vascular endothelial growth factor (*VEGF*), which are important genes in invasion and angiogenesis 40-44. In our study, various changes in proliferation, invasion, migration, and apoptosis were observed in Zwint-1-deficient PC cells, suggesting a role for NF-κB signaling. Zwint-1-deficient PC cells showed reduced phosphorylation of NF-κB and significantly reduced the protein expression levels of NF-κB target genes, including cyclin D1, cIAP1/2, XIAP, MMP2, and MMP9. Consistently, Zwint-1-deficient PC cells also showed reduced proliferation ability, invasion, and migration, as well as increased apoptosis.

## Conclusion

Zwint-1 deficiency inhibits PC cell proliferation, invasion, and migration through the NF-κB signaling pathway, in addition to promoting cellular apoptosis through a caspase-dependent pathway.

## Supplementary Material

Supplementary figures.
