# Supplementary material for: Involvement of the NF-κB signaling pathway in proliferation and invasion inhibited by Zwint-1 deficiency in Pancreatic Cancer Cells
Source: J Cancer. 2020 Jul 25;11(19):5601–11. doi: 10.7150/jca.46173 (PMC7477444; doi:10.7150/jca.46173)
Supplement: Supplementary file 1 — Supplementary figures. [file jcav11p5601s1.pdf]

Involvement of the NF-κB signaling pathway in proliferation and invasion inhibited by Zwint-1 deficiency in pancreatic cancer cells

Jae Hyeong Kim[1], Yuna Youn[1], Jong-chan Lee[1,2], Jaihwan Kim[1], Jin-Hyeok Hwang[1,2*]

[1] Department of Internal Medicine, Seoul National University Bundang Hospital, Seongnam-si, Gyeonggi-do, 13620, Republic of Korea

[2] Department of Internal Medicine, Seoul National University College of Medicine, Seoul, 03080, Republic of Korea

A

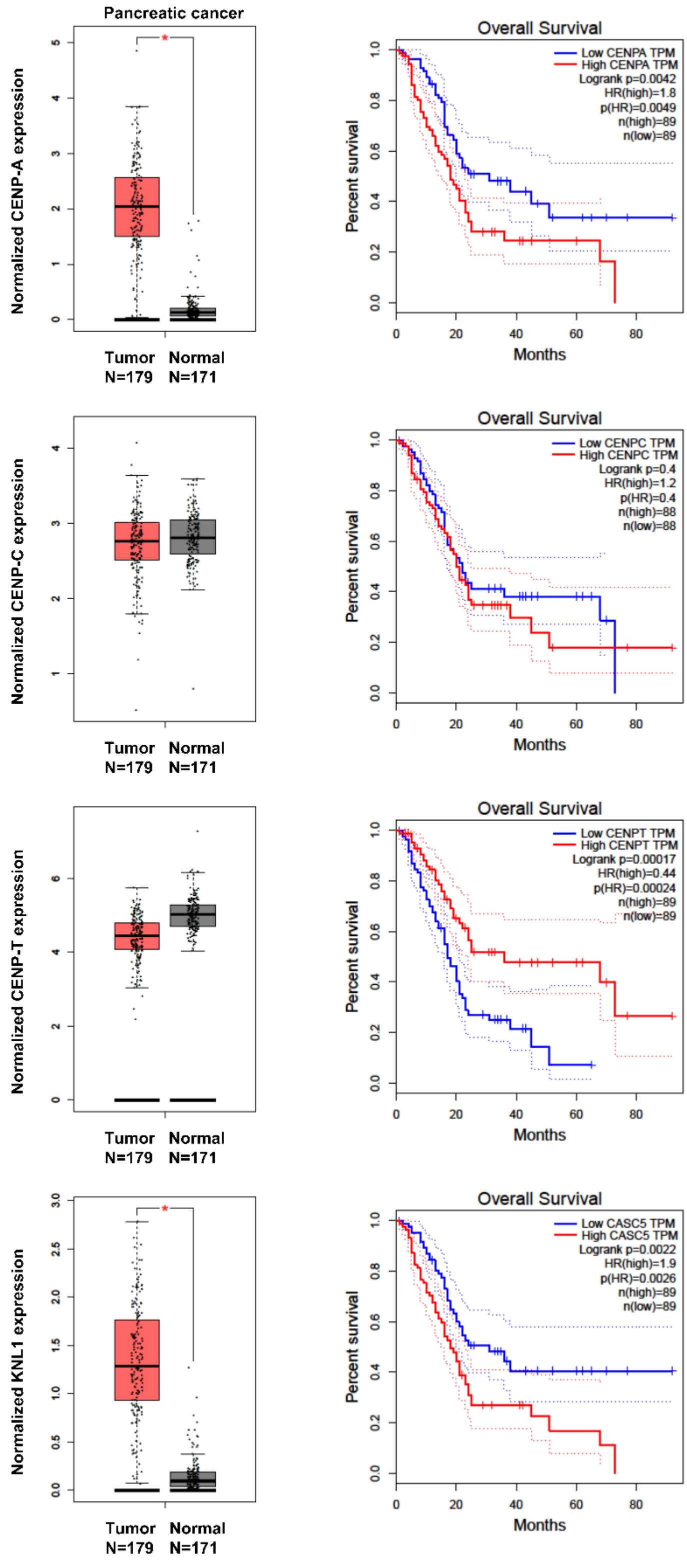
Pancreatic cancer
Normalized CENP-A expression
Tumor N=179
Normal N=171
Overall Survival
Low CENPA TPM
High CENPA TPM
Logrank p=0.0042
HR(high)=1.8
p(HR)=0.0049
n(high)=89
n(low)=89
Percent survival
Months
Normalized CENP-C expression
Tumor N=179
Normal N=171
Overall Survival
Low CENPC TPM
High CENPC TPM
Logrank p=0.4
HR(high)=1.2
p(HR)=0.4
n(high)=88
n(low)=88
Percent survival
Months
Normalized CENP-T expression
Tumor N=179
Normal N=171
Overall Survival
Low CENPT TPM
High CENPT TPM
Logrank p=0.00017
HR(high)=0.44
p(HR)=0.00024
n(high)=89
n(low)=89
Percent survival
Months
Normalized KNL1 expression
Tumor N=179
Normal N=171
Overall Survival
Low CASC5 TPM
High CASC5 TPM
Logrank p=0.0022
HR(high)=1.9
p(HR)=0.0026
n(high)=89
n(low)=89
Percent survival
Months

A

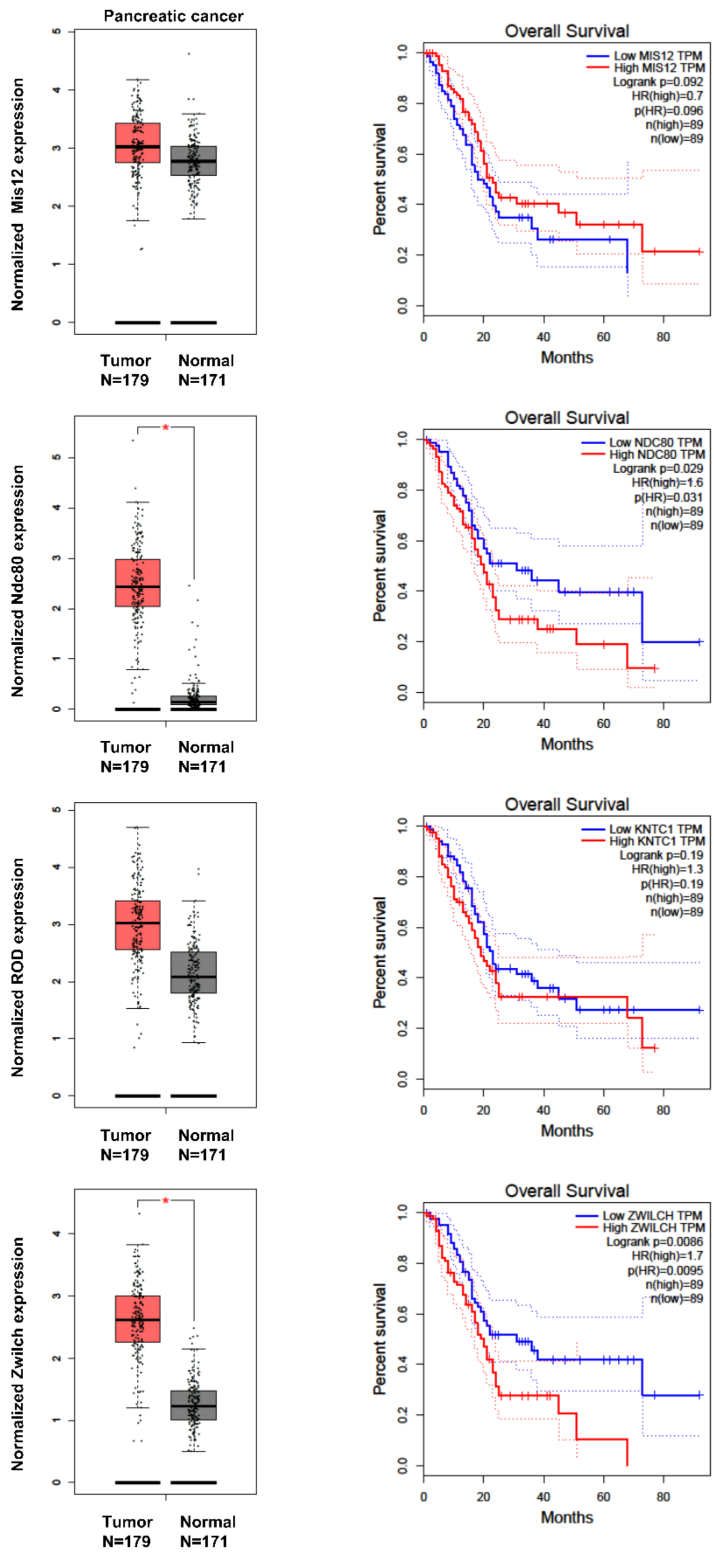
Pancreatic cancer
Normalized Mis12 expression
Tumor
N=179
Normal
N=171
Overall Survival
Low MIS12 TPM
High MIS12 TPM
Logrank p=0.092
HR(high)=0.7
p(HR)=0.096
n(high)=89
n(low)=89
Percent survival
Months
Normalized Ndc80 expression
Tumor
N=179
Normal
N=171
Overall Survival
Low NDC80 TPM
High NDC80 TPM
Logrank p=0.029
HR(high)=1.6
p(HR)=0.031
n(high)=89
n(low)=89
Percent survival
Months
Normalized ROD expression
Tumor
N=179
Normal
N=171
Overall Survival
Low KNTC1 TPM
High KNTC1 TPM
Logrank p=0.19
HR(high)=1.3
p(HR)=0.19
n(high)=89
n(low)=89
Percent survival
Months
Normalized Zwilch expression
Tumor
N=179
Normal
N=171
Overall Survival
Low ZWILCH TPM
High ZWILCH TPM
Logrank p=0.0086
HR(high)=1.7
p(HR)=0.0095
n(high)=89
n(low)=89
Percent survival
Months

A

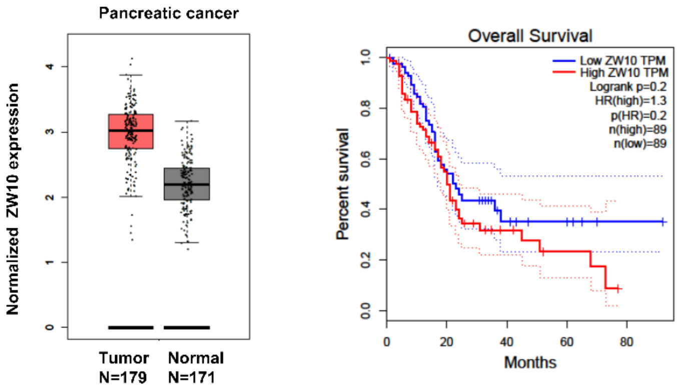

Supplementary Figure 1. Expression of CCAN, KMN, and RZZ complexes in PC tissues and overall survival based on GEPIA data.

(A) Expression levels of CCAN, KMN, and RZZ complexes in PC tissues (n = 179) and normal tissues (n = 171), and overall survival (OS) based on the TCGA database. Each dot represents the expression level of an individual sample. OS of patients with low or high expression levels of subunits of CCAN, KMN, and RZZ complexes was analyzed by the Kaplan–Meier method and log-rank tests. *$P < 0.005$.

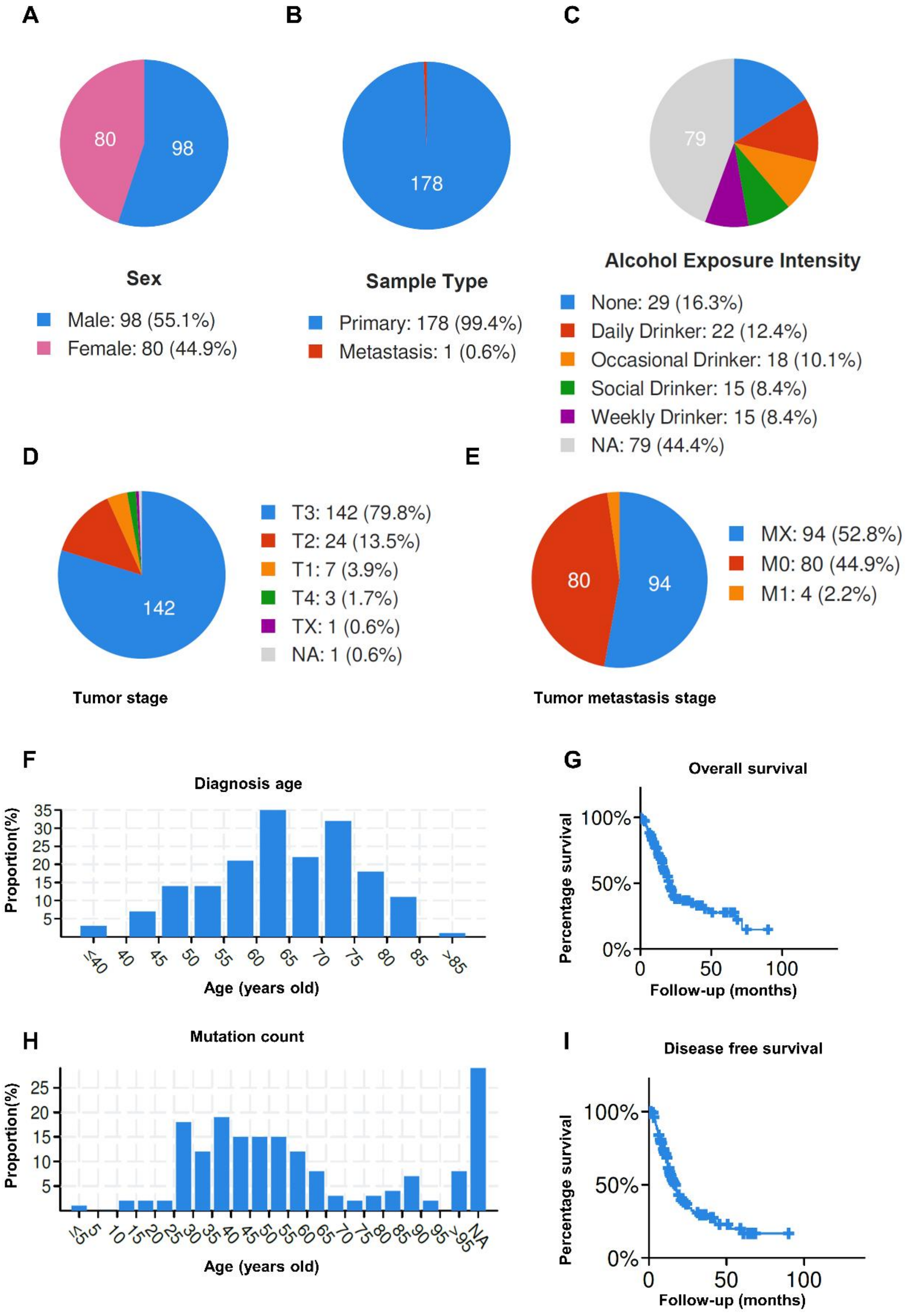
A
B
C
80
98
178
79
Sex
Male: 98 (55.1%)
Female: 80 (44.9%)
Sample Type
Primary: 178 (99.4%)
Metastasis: 1 (0.6%)
Alcohol Exposure Intensity
None: 29 (16.3%)
Daily Drinker: 22 (12.4%)
Occasional Drinker: 18 (10.1%)
Social Drinker: 15 (8.4%)
Weekly Drinker: 15 (8.4%)
NA: 79 (44.4%)
D
E
142
T3: 142 (79.8%)
T2: 24 (13.5%)
T1: 7 (3.9%)
T4: 3 (1.7%)
TX: 1 (0.6%)
NA: 1 (0.6%)
Tumor stage
80
94
MX: 94 (52.8%)
M0: 80 (44.9%)
M1: 4 (2.2%)
Tumor metastasis stage
F
Diagnosis age
Proportion(%)
35
30
25
20
15
10
5
≤40
40
45
50
55
60
65
70
75
80
85
>85
Age (years old)
G
Overall survival
Percentage survival
100%
50%
0%
0
50
100
Follow-up (months)
H
Mutation count
Proportion(%)
25
20
15
10
5
Age (years old)
I
Disease free survival
Percentage survival
100%
50%
0%
0
50
100
Follow-up (months)

Supplementary Figure 2. Clinical characteristics of PC patients in TCGA data.

(A) Sex of patients from the experimental cohort. **(B)** Categorization of patients by sample type (i.e., primary, metastasis). (**C)** Categorization of patients by alcohol exposure intensity. (**D)** Categorization of patients by tumor stage based on the American Joint Committee on Cancer (AJCC). Code of pathological T (primary tumor) to define the size or contiguous extension of the primary tumor (T), using staging criteria from the AJCC. **(E)** Categorization of patients by metastasis stage from the AJCC. Code to represent the defined absence or presence of distant spread or metastases (M) to locations via vascular channels or lymphatics beyond the regional lymph nodes, using criteria established by the AJCC. **(F)** Categorization of patients in the experimental cohort by age at diagnosis. **(G)** Overall survival of patients from the experimental cohort. **(H)** Mutation count of patients from the experimental cohort. (**I)** Disease-free survival of patients from the experimental cohort calcuated from the time of initial treatment.

A

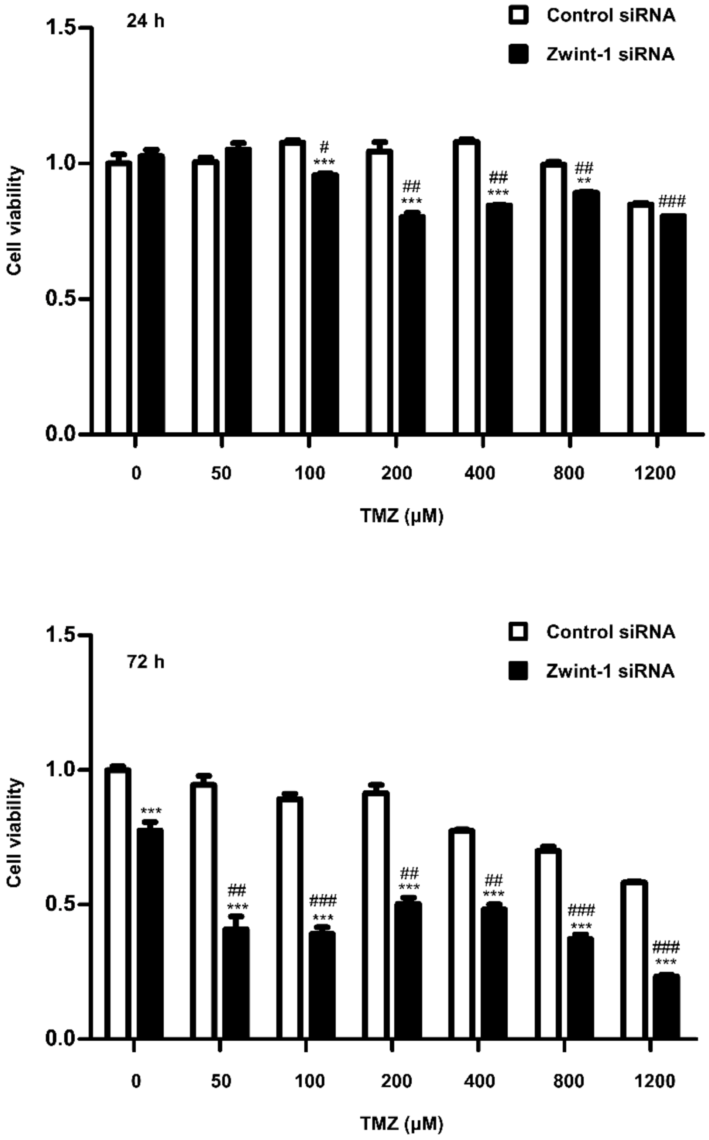

Supplementary Figure 3. Cytotoxic effect of temozolomide (TMZ) in Zwint-1-deficient PC cells.

MIA PaCa-2 cells were transfected with control or Zwint-1 siRNA. Cell viability was determined using the MTT assay after treatment with various concentration of TMZ (50–1200 μM) and culture for **(A)** 24 h and **(B)** 72 h. Data are presented as means ± SEMs of biological

triplicates. $^{**}P < 0.001$, $^{***}P < 0.0001$ vs Control group. Results were analyzed by two-way analysis of variance with Bonferroni's multiple comparison tests. $^{\#\#}P < 0.001$, $^{\#\#\#}P < 0.0001$ vs Zwint-1 siRNA group. Results were analyzed using unpaired *t*-tests.
